# Fluorescent Affibody Molecule Administered *In Vivo* at a Microdose Level Labels EGFR Expressing Glioma Tumor Regions

**DOI:** 10.1007/s11307-016-0980-7

**Published:** 2016-07-05

**Authors:** Ana Luiza Ribeiro de Souza, Kayla Marra, Jason Gunn, Kimberley S. Samkoe, P. Jack Hoopes, Joachim Feldwisch, Keith D. Paulsen, Brian W. Pogue

**Affiliations:** 1 Thayer School of Engineering, Dartmouth College, 14 Engineering Drive, Hanover, NH 03755 USA; 2 CAPES Foundation, Ministry of Education of Brazil, Brasilia, DF 70040-020 Brazil; 3 Department of Surgery, Geisel School of Medicine, Dartmouth College, Lebanon, NH 03756 USA; 4 Affibody AB, SE-171 69 Solna, Sweden

**Keywords:** Pre-GMP ABY-029, Anti-EGFR affibody molecule, Glioma, fluorescence-guided surgery

## Abstract

**Purpose:**

Fluorescence guidance in surgical oncology provides the potential to realize enhanced molecular tumor contrast with dedicated targeted tracers, potentially with a microdose injection level. For most glioma tumors, the blood brain barrier is compromised allowing some exogenous drug/molecule delivery and accumulation for imaging. The aberrant overexpression and/or activation of epidermal growth factor receptor (EGFR) is associated with many types of cancers, including glioblastoma, and so the use of a near-infrared (NIR) fluorescent molecule targeted to the EGFR receptor provides the potential for improving tumor contrast during surgery. Fluorescently labeled affibody molecule (ABY-029) has high EGFR affinity and high potential specificity with reasonably fast plasma clearance. In this study, ABY-29 was evaluated in glioma versus normal brain uptake from intravenous injection at a range of doses, down to a microdose injection level.

**Procedure:**

Nude rats were inoculated with the U251 human glioma cell line in the brain. Tumors were allowed to grow for 3–4 weeks. ABY-029 fluorescence *ex vivo* imaging of brain slices was acquired at different time points (1–48 h) and varying injection doses from 25 to 122 μg/kg (from human protein microdose equivalent to five times microdose levels).

**Results:**

The tumor was most clearly visualized at 1-h post-injection with 8- to 16-fold average contrast relative to normal brain. However, the tumor still could be identified after 48 h. In all cases, the ABY-029 fluorescence appeared to localize preferentially in EGFR-positive regions. Increasing the injected dose from a microdose level to five times, a microdose level increased the signal by 10-fold, and the contrast was from 8 to 16, showing that there was value in doses slightly higher than the microdose restriction. Normal tissue uptake was found to be affected by the tumor size, indicating that edema was a likely factor affecting the expected tumor to normal tissue contrast.

**Conclusion:**

These results suggest that the NIR-labeled affibody molecules provide an excellent potential to increase surgical visualization of EGFR-positive tumor regions.

## Introduction

Glioblastoma is the most aggressive and the most common type of brain malignant tumors in adults with an average survival of 12 months after diagnosis. This poor prognosis is due to the fast growth and highly infiltrative feature of glioma cells, which are able to migrate from the main tumor mass and invade the normal brain parenchyma [1–4]. Preoperative imaging does not always clearly define the edge of the tumor because of this infiltration, and the presence of a positive margin after surgery contributes to frequent recurrence rates [5]. In this study a new contrast agent consisting of a fluorescently labeled Affibody molecule that binds to the epidermal growth factor receptor (EGFR) was examined for uptake in orthotopic glioma tumors to assess the contrast available to guide surgery.

Several recent studies have demonstrated the use of intraoperative fluorescent imaging as a tool to overcome some limitations of conventional white light surgery, to enhance the visual contrast between tumor and normal tissue [6–10]. Oral administration of 5-ALA induces PpIX fluorescence in high grade glioblastoma tumors, which can be imaged during fluorescent-guided surgery (FGS). This has been adopted clinically in several countries, following success reported in both preclinical and clinical trials. However, the observed PpIX accumulation is thought to be partially due to the blood brain barrier breakdown, allowing the ALA to leak through, which can be less pronounced in the tumor edges [11, 12]. In comparison, exogenous protein targeting has allowed identification of several biomarkers which are overexpressed in many types of cancer such as the cell surface receptors EGFR and VEGFR [13–17] which have been studied as targets for therapeutic drug delivery [18–20] and diagnostic imaging [21–23]. Rosenthal and collaborators (2015) described the results of the first-in-human clinical trial using cetuximab conjugated to IRDye® 800CW to guide the surgery of head and neck squamous cell carcinoma and the fluorescence was correlated to EGFR expression of the tumor. The tumor-to-background contrast was dose-dependent, although there was evidence of receptor saturation at higher dose. However, because of the long biological half-life of antibodies, the tumor-to-normal contrast increases over time [24].

In this study, fluorescently labeled affibody molecules (Affibody, Solna, Sweden) targeting EGFR have been developed for human use. Affibody molecules are small proteins (derived from a 58 amino acids long Z-domain scaffold) [25]. The EGRF-binding affibody molecules were engineered with a maleimide linker for site-specific cysteine conjugation with a fluorescent dye such that fluorophore labeling does not interfere with the binding domain site for a receptor, thereby maintaining binding affinity. These molecules have high affinity (approximately *K*_*d*_ ~ 2.8 nM) and demonstrate localization in glioma tumors [13, 26]. Due to their small size, affibody molecules present fast clearance from the blood and reasonable diffusion through tissue, which are the desirable features for a high-contrast surgical imaging agent [25].

In a study performed by Lee and colleagues, the tumor accumulation of an anti-HER2 affibody conjugated to Alexa Fluor® 750 was evaluated. The results showed its potential as a probe for *in vivo* imaging of HER2-positive breast cancer [27]. In a previous study, Sexton et al. showed that the accumulation of an anti-EGFR affibody, conjugated to IRDye® 800CW, was higher in rodent glioma tumors than the anti-EGFR antibody cetuximab conjugated to IRDye® 680RD. The anti-EGFR affibody molecule was present in the tumor periphery whereas the anti-EGFR antibody was primarily localized in the central portion of the tumor [26]. In the present study, the tumor uptake of pre-GMP ABY-029 was evaluated for the contrast in tumor-to-normal brain, as a function of time and administered dose.

## Experimental Session

### Reagents

Anti-EGFR affibody molecules were manufactured under contract from Affibody AB (Solna, Sweden); IRDye® 800CW maleimide was specially produced by LI-COR Biosciences (Lincoln, Nebraska). The anti-EGFR affibody molecules labeled with IRDye® 800CW have been developed by Bachem and named pre-GMP ABY-029.

### Cell Culture

The human glioblastoma cell line U251 was obtained from Dr. Mark Israel (Norris Cotton Cancer Center, Dartmouth-Hitchcock Medical Center, Lebanon, NH, USA) and cultured in DMEM supplemented with 10 % fetal bovine serum and 100 IU/ml penicillin-streptomycin. The cells were subcultivated at 80–90 % of confluence.

### Orthotopic Implantation in Nude Rats

All animal studies were approved by Dartmouth College Institutional Animal Care and Use Committee (IACUC) and conducted in accordance with all institutional Public Health Service (PHS) and Office of Laboratory Animal Welfare (OLAW) guidelines.

Forty-eight female nude rats (6–8-week old) were used. The U251 cell line was chosen due to its clinically relevant level of EGFR expression. The animals were anesthetized using isoflurane (2 % and 1 l/min oxygen) and an incision was made in the scalp, with the brain accessed by a 1-mm rotary drill to create a burr hole. Guided using a stereotaxic frame (Stoelting Co, Wood Dale, IL, USA), the 1 × 10^6^ cells in 5 μl phosphate-buffered saline (PBS) were injected at a 3-mm depth into the left cerebral hemisphere of the rats, 3 mm posterior to the bregma, using a Hamilton syringe (Hamilton Company, Reno, NV). The cells were injected over a 5-min period, and the needle slowly retracted from the brain. Bone wax (Ethicon, Inc., Piscataway, NJ, USA) was used to close the hole in the skull and the incision in the scalp closed, using 5–0 sterile non-absorbable suture material (Ethicon, Inc., Piscataway, NJ, USA).

A control group received an identical procedure without tumor cells (saline only).

### Magnetic Resonance Imaging

Contrast-enhanced magnetic resonance imaging was used to monitor tumor growth in the brain. In these studies, the rats were anesthetized with isoflurane and maintained at a surgical anesthesia plane through the procedure. A Phillips Achieva 3.0T X-series MRI with a modified rodent coil (Philips Research Europe, Hamburg, Germany) was used [23]. T1- and T2-weighted turbo-spin echo images were acquired prior to intravenous administration of 0.1 mmol of gadolinium Gd-DTPA (Magnevist®) and the post-contrast T1W image sequence was collected 10 min following gadolinium injection. The T2W image sequence was collected during the contrast uptake. The MRI images were processed and analyzed using NIRFAST Software (Version 1.12) [28, 29].

### Fluorescent Tracer Imaging Experiments

Two weeks post-tumor cell inoculation, the rats received a non-fluorescent diet (Purified Mouse Diet, MP Biomedicals, LLC, Illkirch, France) to reduce auto-fluorescence from chlorophyll contained in regular chow. Three to 4 weeks post-tumor inoculation, rats were randomly divided into 10 experimental groups to either receive different doses of ABY-029 (24.5, 49.0, or 122.5 μg/kg) at different time points (1–48 h) or PBS (control group). The lowest dose, 24.5 μg/kg, corresponds to the human equivalent microdose for a protein molecule, defined by the FDA as ≤30 nanomoles (Guidance for Industry, Investigators, and Reviewers-Exploratory IND Studies, 2006). After the predetermined time, the animals were euthanized by cervical dislocation; the brain was removed and sectioned into 2-mm thick slices. Three U251 inoculated rats and two sham rats, for each group, were used.

Fluorescence images from the sequential brain sections were acquired by scanning the 2-mm thick slices on the Odyssey Infrared Imaging System (LI-COR Biosciences) using the 800-nm channel, at 21-μm lateral spatial resolution. Eight to ten slices from each rat were examined in a single scan, and completed within minutes of brain removal.

For fluorescence image contrast analysis, the relative difference between the fluorescence intensity in the tumor and normal brain tissue was calculated according to the equation:

$$ \mathrm{Contrast}=\frac{\left({F}_{\mathrm{T}}-{F}_{\mathrm{N}}\right)}{\left({F}_{\mathrm{N}}-{F}_{\mathrm{B}}\right)} $$

where *F* is the region of interest (ROI) fluorescence from tumor (*F*_T_) and contralateral normal (*F*_N_) brain quantified in ImageJ® software (NIH, Bethesda, MD), and background being outside the brain region (*F*_B_). The ROI of both tumor region and contralateral normal brain was guided by custom delineation based upon the fluorescence images and confirmed with the hematoxylin and eosin images.

### Hematoxylin and eosin and Immunohistochemistry Staining and Microscopy Analysis

Following analysis of the fluorescence in the brain sections, the samples were fixed overnight in 4 % neutral buffered formaldehyde. Research Pathology Services at the Geisel School of Medicine at Dartmouth College prepared the following histopathology and immunohistochemistry tissue sections. Formalin-fixed tissues were dehydrated using crescent concentrations (70–100 %) of alcohol (Fisher Scientific, USA), cleared with xylene (Cat # X3P-1GAL, Fisher Scientific, USA) and embedded in paraffin (Cat # 7052, Sakura Finetek, Torrance, CA, USA). The brain sections were cut to 4-μm thickness and stained with hematoxylin and eosin (H&E) and for total EGFR using an anti-EGFR primary antibody (EP38Y) (Cat # ab52894; Abcam Inc., Cambridge, MA, USA) (immunohistochemistry).

Whole brain images were acquired using a light microscope (Zeiss, Stemi SV 11 APO) at ×0.6 magnification. ROI of tumor and normal tissue were analyzed at ×10 magnification (Olympus-BX50).

### Statistical Analysis

Statistical analysis was performed using OriginPro® 8 software (OriginLab, Wellesley Hills, MA, USA). Student’s paired *t* test was performed to determine statistical significance of the difference in signal between the tumor regions and corresponding contralateral brain or animals undergoing sham surgery. Statistics assessment was also used to determine the difference between different doses and time points. Linear regression was used to compare the size of the tumor and contralateral normal brain fluorescence. This information was reported as a Pearson correlation coefficient (*r*). Results were considered statistically significant at a *P* < 0.05.

## Results

**Fig. 1.**
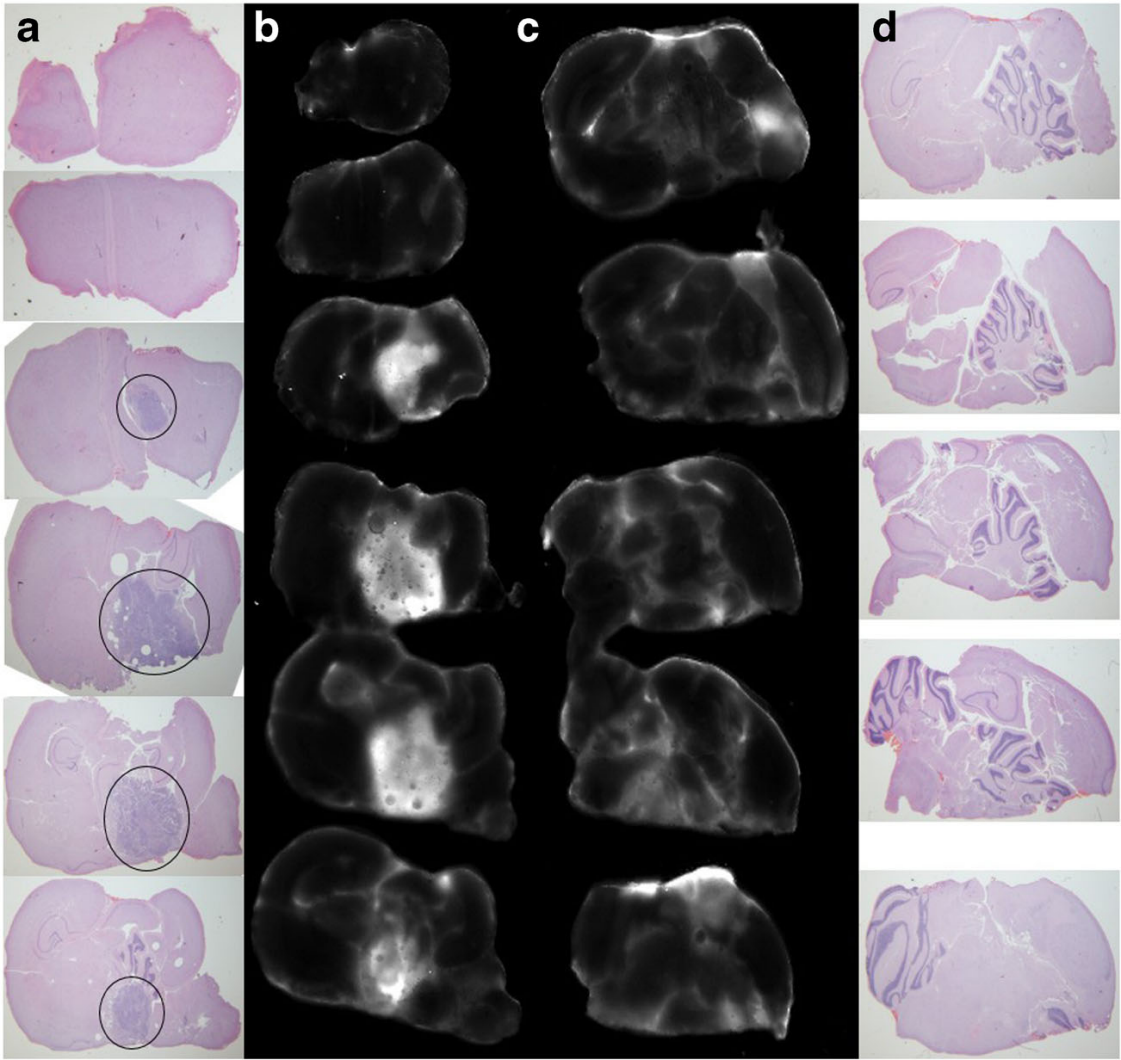
Multiple plane co-registration of brain tumor morphology (**a**, **d**) and ABY-029 fluorescence (**b**, **c**) in a series of brain slices from the same rat. The tumor regions are fairly obvious in the H&E, and are *circled* in the *lower left H&E images*, which correlate well with the fluorescence areas. Each row from columns **a** and **b** or **c** and **d** correspond to the same slice of the brain

Pre-GMP ABY029 uptake was evaluated using an infrared imaging system and the level of fluorescence from rat *ex vivo* brain slices was acquired at different time points post-injection. Figure 1 presents the 1:1 correlation of whole brain fluorescence and H&E histologic staining. This co-registration demonstrates a high correlation between tumor presence and fluorescence.

**Fig. 2.**
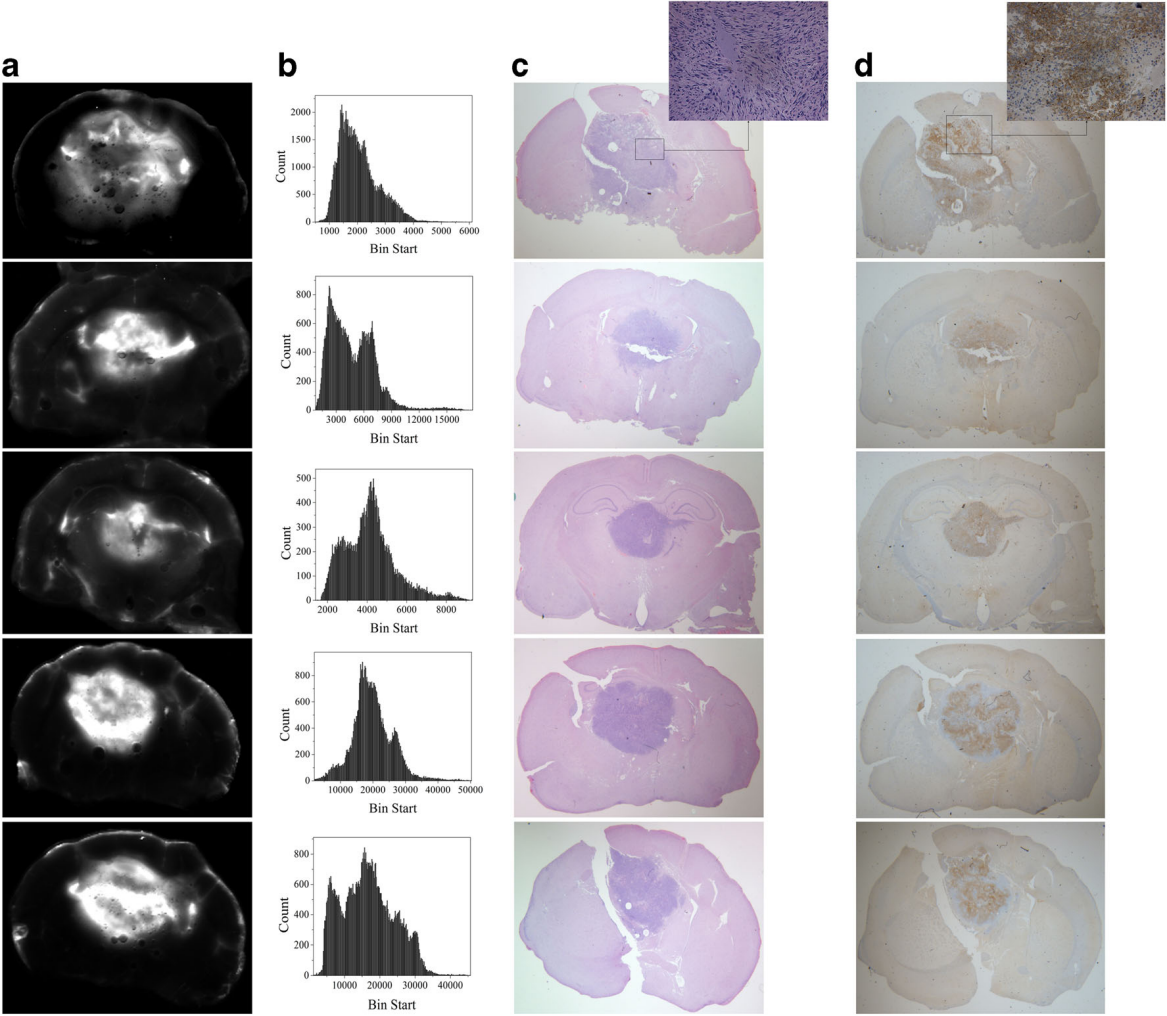
**a** The distribution of ABY-029 fluorescence in the tumor region immediately after tissue removal. **b** The heterogeneity of fluorescence signal distribution for each corresponding slice. **c** The H&E-stained tissue. **d** The EGFR-stained tissue. This information illustrates the true EGFR expression patterns.

There was high fluorescence heterogeneity, within and around the tumors (Fig. 2a). Histograms of the tumor fluorescence, shown in Fig. 2b, demonstrate the regular presence of two regions of intensity. The regions of high ABY-029 fluorescence intensity were often well correlated to the regions of high EGFR expression, as illustrated by *ex vivo* staining using anti-EGFR antibodies in Fig. 2d. The EGFR-specific staining confirms the localization patterns expected; however, it is important to note that low level ABY-029 fluorescence is still visible in the regions with lower EGFR-specific staining with antibodies.

**Fig. 3.**
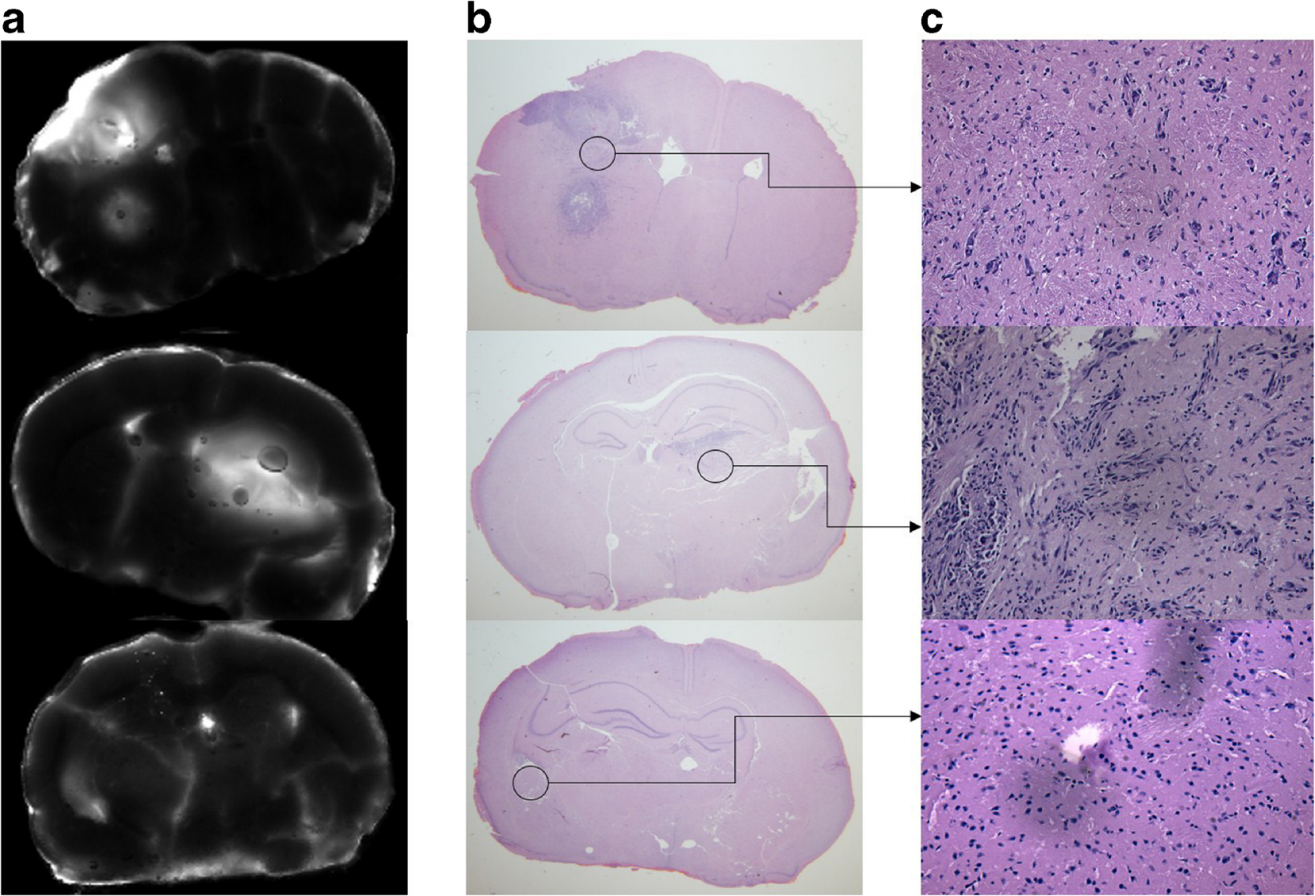
These images represent the presence of fluorescent (EGFR) tumor cells at the margin of the primary tumor mass. **a** *Ex vivo* fluorescence analysis. **b** H&E staining (×0.6 magnification). **c** H&E staining (×10 magnification) and visualization of tumor cells migrating into normal brain tissue. The last row corresponds to sham surgery and, therefore, serves as a control brain tissue.

Another feature evaluated in this study was the ability of ABY-029 to recognize positive margins in which migratory cells are presented (Fig. 3). Tumor cells cannot only migrate through blood vessels to infiltrate the normal brain, but also can be derived from the main tumor mass and migrate in surrounding brain tissue as seen by ABY-029 fluorescence in Fig. 3a.

**Fig. 4.**
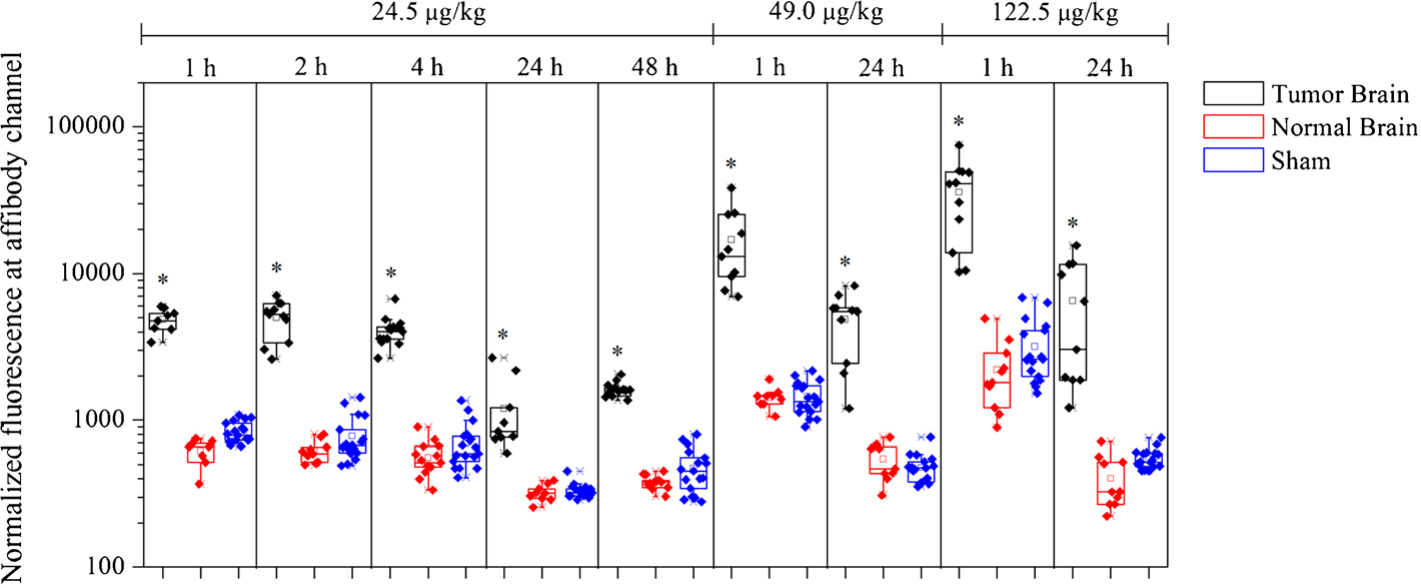
Normalized fluorescent signals are shown for all tumor and normal tissues slices analyzed, at all three dose levels (24.5, 49, 122.5 μg/kg), and at varying times following injection (1 h up to 24 h or 48 h). *P < 0.05, Student’s *t* test.

The effect of dose escalation and temporal analysis of the fluorescence is demonstrated in Fig. 4. The peak of fluorescence in tumors is observed at early times (1–4 h) in a dose-dependent manner and there is significant fluorescence in the glioma tumors for several hours after injection. Moreover, fluorescence in the tumor regions was significantly higher than in the contralateral normal brain, or in the brains of rats that received a sham surgery.

**Fig. 5.**
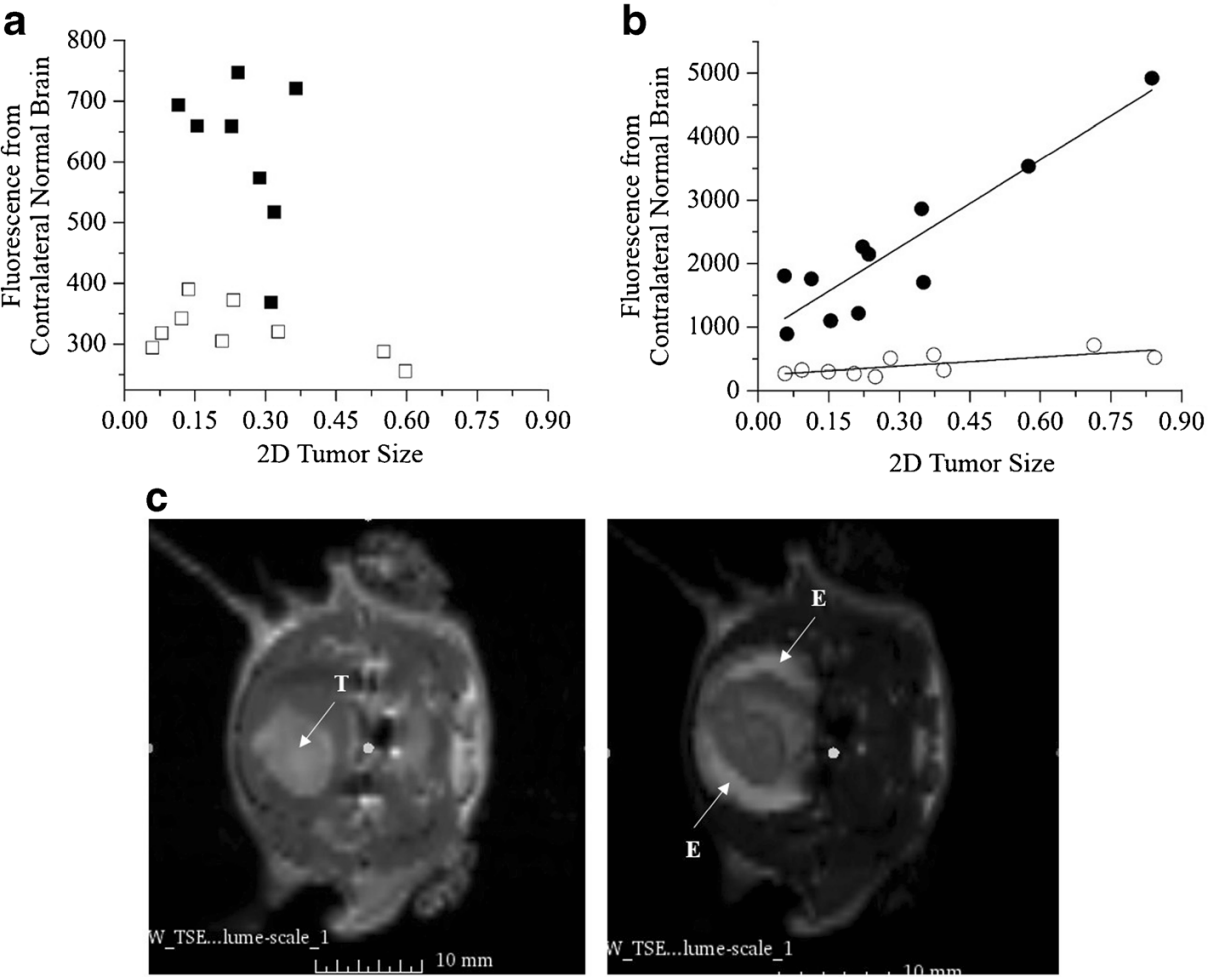
Fluorescence from the contralateral normal brain as a function of the observed tumor size for **a** microdose (24.5 μg/kg) and **b** five times the microdose (122.5 μg/kg); 1 h (*closed symbols*) and 24 h (open symbols) post-injection. The lines correspond significant trends observed from linear regression. **c** T1 (*left*) and T2 (*right*) MRI of a U251 tumor-bearing rat post-gadolinium injection. The tumor (*T*) can be seen as a focal region in the T1, with peri-tumor edema (*E*) in the T2 image.

Figure 5a, b presents the fluorescence signal intensity from contralateral normal brain as a function of the tumor area detected by the lowest and highest dose of the pre-GMP ABY-029. There are no statistically significant fluorescence trends for tumor size in the 1× microdose group; however, there is a significance when the dose is elevated fivefold.

A strong positive linear correlation (*r* = 0.91 and *r* = 0.74 for 1 h and 24 h post-injection, respectively) (*P* < 0.05 for Pearson correlation) was determined to five times the microdose, likely from edema in the normal brain (Fig. 5c).

**Fig. 6.**
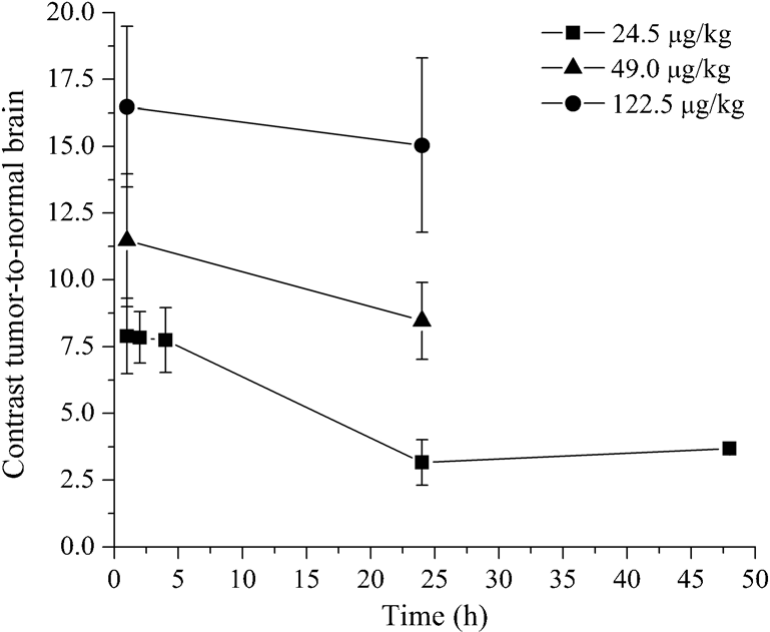
The tumor-to-normal brain contrast values are expressed as mean values ± standard error of mean using the ratio of data from Fig. 4 for each injection concentration and time point studied.

The success of a fluorescence-guided brain surgery is highly dependent of the tumor-to-normal brain fluorescence contrast, and the results are presented in Fig. 6. The contrast relative to the normal brain is dependent on the dose given and the time of data acquisition after dose administration. In our study, the contrast varied from 7 to 16 in the first 4 h. After 24 h, in the microdose level, the contrast is reduced by a factor of 3 to 2.7 resulting in a very low fluorescence intensity. However, with higher administered ABY-029 doses (two or five the microdose level), the contrast level decreased only slightly over time.

## Discussion

Several published studies have tested the concept of a receptor-binding molecule, conjugated to a near-infrared fluorescent probe that is able to be localized in the margin of tumor and normal tissue. The goal of this and similar research is to increase the tumor-to-background contrast ratio as a means of improving the tumor delineation during surgical resection, and to determine if injection at the microdose level was a reasonable administration concentration to achieve this. In 2011, van Dam and colleagues studied the use of folate conjugated to fluorescein isothiocyanate (FITC) to target the folate receptor α (FR-α) overexpressed in ovarian cancer. This receptor-targeting fluorescent probe demonstrated a promising approach for detection of the receptor positive human tumors compared to the conventional visual inspection during standard surgery [30]. In neurosurgery, substantial efforts have been made with the metabolic marker protoporphyrin IX produced by mitochondrial heme synthesis [31–34], and indocyanine green perfusion imaging [35–37] to delineate the tumor borders during brain surgery. Based on this basic research, dedicated surgical microscope systems are now commercially available for fluorescence image acquisition in either the red (600–750 nm) or near-infrared (800+ nm) wavelength bands [38].

In this study, the tumor uptake of ABY-029 was examined as a function of its administered dose and time after injection. The co-localization of the tumor microenvironment and Affibody-fluorescent probe signal was observed through bulk analysis (Figs. 1, 2, and 3). However, the distribution of the fluorescence signal in the tumor was also documented to be highly heterogeneous (Fig. 2). This results from cellular heterogeneity and differentiation level of the glioma cells [39], and/or the spatial and temporal heterogeneity of the blood supply, leading to high delivery variability from the enhanced permeability and retention effect [40, 41]. The absence of fluorescence usually was observed in the tumor interior and it could be either correlated with necrotic areas (Fig. 2c inset), in which vascular supply is often inadequate or insufficient, or lower interstitial pressure present in peripheral areas of the tumor that allows leakage of macromolecules [40]. Perhaps most importantly, the EGFR overexpression was documented by *ex vivo* staining with an independent antibody, and the presence of hot spots staining of ABY-029 appeared to be well correlated to the EGFR density (Fig. 2d inset).

Glioma cells are highly infiltrative i.e., they have the ability to migrate from the main tumor mass to the normal tissue surrounding the margins of the tumor. The accurate anatomical delineation of the tumor boundaries during surgery is challenging, and it is extremely important for the clinical success in the surgery. In this study, ABY-029 fluorescence was seen in tumor cells at the margin of main tumor mass (Fig. 3).

One point to be considered is the fact that EGFR is also highly expressed in some normal tissues, such as the liver, skin, and submaxillary salivary gland. Therefore, the bioavailability of ABY-029 could be reduced as a consequence of off-target EGFR binding of the NIR probe. The results from dose escalation showed a dose-dependent ABY-029 tumor uptake (Fig. 4), which can be related to saturation of EGFR in the liver [42]. Moreover, the fluorescence from the tumor was higher than the contralateral normal brain or brain from the rats that received sham surgery. This was true even 48 h post-injection. Thus, these results show that there is high *in vivo* specificity for ABY-029 uptake by tumor cells.

An interesting result observed in this study was the increase in the contralateral normal brain fluorescence as a function of tumor area for the animals that received 5-fold larger dose than the initial dose (Fig. 5b), which could be correlated to the transport, likely due to edema, leading to nearly 10-fold higher uptake in the tumor, and approximately a 2-fold increase in tumor to normal tissue contrast. Curiously, at the lower injected dose of 24.5 μg/kg, the increase in normal brain uptake with tumor size was not as apparent, but this could have been due to the lower leakage rates into the tissue from lower concentration. In the cases of larger tumors, the ABY-029 uptake in the background normal brain presented in the extracellular matrix by the cerebrospinal fluid to intact areas of the brain, as seen in Fig. 5c. Similar results have been documented with PpIX fluorescence imaging near glioma tumors [43]. More importantly, though, this increased background from edema-based perfusion to normal brain did not significantly decrease the tumor˗-to-normal contrast (Fig. 6), and a high contrast could be seen even after 24 h post-injection, which is important to cover all possible times for surgical use.

## Conclusion

ABY-029 can be used in intraoperative fluorescent-guided surgery of glioma in order to improve detection of the tumor-to-normal tissue boundary and decrease the residual tumor cells surrounding the tumor margin, which is the key to improved prognosis of following resection of glioblastoma. The value of increasing the injected dose above a microdose appears clear, providing significant 2-fold increase in contrast and also a longer term increase extending out to 24 h. However, it is possible to see contrast in glioma tumors with injected doses as low as a standard FDA microdose level. GMP production of ABY-029 is in progress, and toxicological studies have been completed to confirm the safety of the molecular-targeted fluorescent probe for future clinical trial [44].
